# Specific Plant Mycorrhizal Responses Are Linked to Mycorrhizal Fungal Species Interactions

**DOI:** 10.3389/fpls.2022.930069

**Published:** 2022-06-10

**Authors:** Xin Guo, Ping Wang, Xinjie Wang, Yaoming Li, Baoming Ji

**Affiliations:** ^1^School of Grassland Science, Beijing Forestry University, Beijing, China; ^2^Command Center for Integrated Natural Resource Survey, China Geological Survey, Beijing, China; ^3^College of Forestry, Beijing Forestry University, Beijing, China

**Keywords:** arbuscular mycorrhiza fungi, mycorrhizal growth response, plant-soil feedback, co-occurrence network, network complexity

## Abstract

Effects of arbuscular mycorrhizal fungi (AMF) on plants span the continuum from mutualism to parasitism due to the plant–AMF specificity, which obscures the utilization of AMF in the restoration of degraded lands. *Caragana korshinskii*, *Hedysarum laeve*, *Caragana microphylla*, *and Poa annua* are the most frequently used plants for revegetation in Kubuqi Desert, China, and the influence of AMF on their re-establishment remains to be explored further. Herein, using a greenhouse experiment, we tested the plant–AMF feedbacks between the four plant species and their conspecific or heterospecific AMF, retrieved from their rhizosphere in the Kubuqi Desert. AMF showed beneficial effects on plant growth for all these plant-AMF pairs. Generally, AMF increased the biomass of *C. korshinskii*, *H. laeve*, *C. microphylla*, *and P. annua* by 97.6, 50.6, 46.5, and 381.1%, respectively, relative to control. In addition, the AMF-plant specificity was detected. *P. annua* grew best, but *C. microphylla* grew worst with conspecific AMF communities. AMF community from *P. annua* showed the largest beneficial effect on all the plants (with biomass increased by 63.9–734.4%), while the AMF community from *C. microphylla* showed the least beneficial effect on all the plants (with biomass increased by 9.9–59.1%), except for *P. annua* (a 292.4% increase in biomass). The magnitude of AMF effects on plant growth was negatively correlated with the complexity of the corresponding AMF co-occurrence networks. Overall, this study suggests that AMF effects on plant growth vary due to plant-AMF specificity. We also observed the broad-spectrum benefits of the native AMF from *P. annua*, which indicates its potential utilization in the restoration of the desert vegetation.

## Introduction

Arbuscular mycorrhizal fungi (AMF) are common rhizosphere microorganisms, which are in a mutually beneficial symbiotic relationship with up to 80% of terrestrial plant species (Wang and Qiu, 2006). By forming a biotrophic symbiosis with plants, AMF could enhance the plant uptake of relatively immobile nutrients (Chandrasekaran, 2020). Yet, functional diversity among AMF exists because they come from different guilds with distinct traits. Based on the colonization strategy and the phylogeny of AMF (Hart and Reader, 2002; Maherali and Klironomos, 2007), Weber et al. (2019) classified AMF into three guilds: the ancestral, edaphophilic, and rhizophilic AMF. Accordingly, the edaphophilic AMF with high biomass allocation to extraradical hyphae favored nutrient uptake, while rhizophilic AMF with a high allocation to intraradical hyphae favored pathogen protection. In addition, they also differed in their response to environmental changes (Han et al., 2020; Babalola et al., 2022). Thus, the reintroduction of AMF in degraded lands could decrease the mortality of seedlings by improving nutrient uptake and counteracting the Janzen–Connell effect of soil pathogens, promote the establishment of pioneer and later successional plant species, and drive the succession of grassland ecosystems (Liang et al., 2015; Koziol et al., 2018; Neuenkamp et al., 2019).

However, the costs and benefits of maintaining a symbiosis with AMF differ significantly for plants, resulting in varied plant growth response to AMF (Feddermann et al., 2010; Mensah et al., 2015; Monier et al., 2017). Similarly, plant species can respond differently to distinct AMF species due to differed AMF characteristics (Wilson and Hartnett, 1998; Yang et al., 2014; Ramirez-Viga et al., 2018). Thus, the plant–AMF interactions are always not positive, but range from positive to neutral and even negative (Pankova et al., 2018; Neuenkamp et al., 2019). For example, Kaur et al. (2022) inoculated two sorghum accessions with two AMF species (*Rhizophagus irregularis* and *Gigaspora gigantea*) and observed both positive and negative effects. He then found that the positive outcome of *R. irregularis* was associated with a faciliatory root metabolome, while the higher relative abundance of sugars in the *G. gigantea* treatment may indicate a potential carbon drain and lead to negative effects. Bennett and Groten (2022) further concluded that the interaction between a plant and AMF was context-dependent and could be influenced by environmental changes, such as nutrient availability and abiotic stress. In addition, biotic interactions between plants and other microbes, such as pathogens, would also influence the plant-AMF relationships (Berendsen et al., 2012; Bennett and Groten, 2022).

To decrease such AMF uncertainties in restoration activities, researchers have tried to understand the factors influencing plant–AMF interactions and to find the optimal plant–AMF matches (Herrera-Peraza et al., 2011; Rua et al., 2016; Yang et al., 2018; Johnson and Gibson, 2020; Guisande-Collazo et al., 2022). According to the well-established plant–soil feedback theory, one plant species could specifically recruit the microbes beneath it (Bever, 1994; Bever et al., 1997; van der Putten et al., 2013). For example, Tsiknia et al. (2021) found that host plant identity was the major driver of AMF community in the plant roots in the Mediterranean sand dunes. Sepp et al. (2019) also reported the significant effects of host plant species and host plant functional groups on AMF richness and community composition in a biodiverse semi-natural grassland. He further found a larger-than-random degree of selectivity of AMF among plants by network analysis. Even closely related plant species had dissimilar AMF communities (Veresoglou and Rillig, 2014). Moreover, previous research reported that host plants could specifically select fungal symbionts and result in unique AMF network topologies and community structures (Sepp et al., 2019). Such specific effects were because both the host plants and AMF could preferentially allocate resources to higher quality partners (Bever et al., 2009; Bever, 2015; Ji et al., 2016) and consequently achieve optimal plant-AMF matches or plant-AMF specificity (Klironomos, 2003; Ji et al., 2010, 2013; Johnson et al., 2010). However, the effects of such species-specific AMF on neighboring plant species would be unpredictable. For instance, Qiao et al. (2016) found increased biomass of maize and faba bean by AMF, while the biomass of a weed species was decreased when grown together. Pizano et al. (2019) conducted a plant–soil feedback experiment with 10 plant species in a fragmented montane agricultural system. He found inconsistent feedback of AMF on several pairs of native species. In addition, Chen et al. (2020) grew one native species together with one invader and found that native plants had reduced AMF colonization and benefits compared to the invader. Muneer et al. (2022) found that the nitrogen transfer by AMF was different for two grass species when grown together. Nevertheless, the mechanisms underlying the plant-AMF specificity are poorly understood, causing high uncertainties in AMF utilization in the restoration of degraded lands (Moora et al., 2004; Pizano et al., 2019; Lubin et al., 2020; Guo et al., 2021).

Grasslands are one of the world’s most important ecosystems, accounting for over one-third of the earth’s terrestrial surface (O’Mara, 2012). However, during the last few decades, natural ecosystems including grasslands suffered from various kinds of degradation owing to anthropogenic activities and global environmental changes (Gang et al., 2014). Although great efforts have been made on the restoration projects, the results have not always been as successful as expected (Koziol et al., 2016; Wubs et al., 2016), partly due to changes in the soil microbiome in degraded grassland soils, particularly the beneficial soil microbes (Requena et al., 2001; Tian et al., 2009; Asmelash et al., 2016; He et al., 2020). *C. korshinskii*, *H. laeve*, *C. microphylla*, and *P. annua* are the common species found in the desert of northern China and are frequently used for revegetation, but their revegetation was still a big challenge (Li et al., 2004). Specifically, the effects of AMF on the growth of *C. korshinskii*, *H. laeve*, *C. microphylla*, and *P. annua* are still not clear.

Herein, we examine the effect of conspecific or heterospecific AMF on the growth of the four species (i.e., *P. annua*, *C. korshinskii, H. laeve*, and *C. microphylla*) in a greenhouse. The conspecific and heterospecific AMF inocula were retrieved from the rhizosphere of these four host species in the desert. We hypothesize that (a) these four native AMF communities would have positive effects on the growth of these four species (i.e., *P. annua*, *C. korshinskii, H. laeve*, and *C. microphylla*); and (b) plants inoculated with conspecific AMF would grow better than those inoculated with heterospecific AMF.

## Materials and Methods

### Inoculum Preparation

In June 2014, plant root and soil samples (both bulk and rhizosphere soil) were collected using a soil auger (5 cm diameter) to the depth of 20 cm, targeting *C. korshinskii* (A), *H. laeve* (B), *C. microphylla* (C), *and P. annua* (D) (one core for each individual and 10 random individuals for each plant species) in the Kubuqi Desert (40°04′47.13″N, 110°46′34.83″E). Desert soil samples (0–20 cm) were also collected simultaneously near the plants and steam-sterilized (121°C, 1 h, twice) to serve as a growth substrate for the following trap culture of AMF. The collected root and soil samples for each of the four species were then separately mixed with the sterilized growth substrate in a 1:2 (v/v) ratio in 10 sterilized plastic pots (2 L volume). Then, each pot was sown with 50 seeds of maize (sterilized with 75% alcohol). All pots were amended with 200 ml of non-sterilized field soil sievates, with 50 ml each from the four species, to equalize microbial communities of the trap culture soils except AMF. In our study, field soil sievates were obtained by blending 1 L of soil with water in a 1:2 ratio and passing the slurry through a 38-μm sieve. In this way, the relatively large AMF spores and hyphae were trapped on the sieve, while smaller organisms passed through. Proposed by Koide and Li (1989), this method was used in many previous studies regarding AMF inoculation (Johnson et al., 2010; Ji et al., 2016; Guo et al., 2021). Then maize plants were grown in a greenhouse in the Inner Mongolia Academy of Forestry at Hohhot and were watered weekly. Three months later, above-ground parts of the maize were removed, and the underground contents were harvested separately and used as inocula for the following experiments. Maize roots were removed from the soil, cut into 1 cm lengths, and then mixed back with the soil. Thus, the inoculum consisted of AMF-colonized root pieces and spores, and hyphae originated from *C. korshinskii* (A), *H. leave* (B), *C. microphylla* (C), and *P. annua* (D), respectively. Five subsamples of the inoculum A, B, C, and D were stored at –20°C for subsequent AMF community analysis, and the remaining soil samples were used in the following plant–AMF feedback experiments. The number of spores in the inoculum was about 300 per 100 g of inoculum as in a previous study (Guo et al., 2021). Notably, though AMF communities could change during the trap culture, the influence was minor considering the relative short-term conditioning time (Ke et al., 2021).

### Experimental Design

The pot experiment was established in a greenhouse at Hohhot in the Inner Mongolia Province and lasted from March to June 2016. The full factorial experiment consisted of two factors including four host species and five AMF inoculation treatments with a replicate of 10. Four host plant species were *C. korshinskii* (A), *H. laeve* (B), *C. microphylla* (C), *and P. annua* (D). AMF inoculation treatments included inocula originated from the four different plant species (AMF inoculum A, B, C, and D were from trap cultures of *C. korshinskii, H. laeve, C. microphylla, and P. annua*, respectively) with sterilized inoculum acting as a control. Thus, the four plant species were fully cross-inoculated with the five AMF inoculation treatments (including control). Overall, there were 200 pots in this experiment (i.e., 4 host species × 5 AMF inoculation × 10 replicates).

Seeds of *C. korshinskii, H. laeve, C. microphylla, and P. annua* were collected in the Kubuqi Desert at the same time as soil sampling. After disinfection with 75% alcohol, these seeds were washed with sterilized tap water, and then pre-germinated in a pot with sterilized growth substrate for 3 weeks. Seedlings with good status were then transplanted to the 1 L plastic pots containing the growth substrate and the corresponding inoculum. The soil texture is arenosol and contained 1.0 g kg^–1^ soil organic carbon, 0.09 g kg^–1^ total N, and 4.36 mg kg^–1^ available P. At the transplanting stage, each 1 L pot was filled with 600 cm^3^ of growth substrate at first. After adding 100 cm^3^ of the corresponding AMF inoculum, three seedlings of the same species were transplanted directly into the inoculum, and then another 200 cm^3^ of growth substrate was used to fill the pot. The uninoculated control received 100 cm^3^ of the sterilized inoculum. In addition, 200 ml of filtered sievates of AMF inocula with 50 ml each from inoculum A, B, C, and D were also added to correct for possible differences in non-AM microbial communities as mentioned in the trap culture above. Then plants were grown for 14 weeks at a temperature of about 25°C in a greenhouse in the Inner Mongolia Academy of Forestry at Hohhot. The pots were watered three times a week and were repositioned randomly once a week to minimize the microclimate variation until harvested in June 2016.

### Measurements and Sample Collection

At harvest, the average plant height of all the individuals in each pot was recorded. The total above-ground biomass of all the individuals in the pot was recorded after oven drying at 70°C for 48 h. The height and above-ground biomass of the plants were similar among the 10 replicates. Thus, four replicate pots from each treatment were randomly selected for AMF colonization measurement. The roots in each selected pot were harvested and gently washed. Then, ten 2-cm root pieces from each pot were picked. Overall, there were 40 root pieces used for AMF colonization measurement for each treatment. These roots were then cleaned in 10% KOH and acidified with 1% HCl. The surface of the root samples was then stained with 0.05% trypan blue in lactophenol (Phillips and Hayman, 1970). AMF colonization was recorded by the magnified gridline intersect method (McGonigle et al., 1990). An additional subset of roots was stored at –20°C for subsequent molecular analyses. The remaining roots were weighed, dried, and then reweighed to produce a wet-to-dry root biomass prediction equation to obtain the total dry root biomass of the samples. Total oven-dried plant weight was then divided by the number of survived seedlings to get individual plant biomass.

### DNA Extraction and Sequencing

Total DNA of 20 soil samples after trap culture (five each from inoculum A, B, C, and D) was extracted using the DNeasy Power Soil Kit (Qiagen, Hilden, Germany) according to the manufacturer’s protocol. After harvesting, the total DNA of inoculated plant roots (three or four replicates for each AMF treatment and host plant combination) was extracted by the CTAB method (Allen et al., 2006). Fresh roots potentially colonized by AMF were cut into 1-cm segments, and 20 fragments of them were picked up for DNA extraction.

Glomeromycotina sequences were amplified by nested PCR with the SSU rRNA gene primers NS31–AML2 and AMV4.5NF–AMDGR (Van Geel et al., 2014) as described in a previous study (Guo et al., 2021). We used the UPARSE pipeline (Edgar, 2013) and the Quantitative Insights Into Microbial Ecology (QIIME v1.7.0, United States) (Caporaso et al., 2010) to treat all raw sequencing data. Briefly, sequences with a quality score lower than 20 were discarded, resulting in 6,146,319 sequences. Among them, 1,024,772 sequences were uniques and 843,909 sequences were singletons (82.4%). Primer-free sequences were dereplicated. Then chimeric sequences were removed using the UCHIME method (Edgar et al., 2011). The resulting non-chimer sequences were binned into operational taxonomic units (OTUs) with 97% similarity by Usearch ver. 11.0 (Edgar, 2010). The most abundant sequence from each OTU was selected as the representative sequence corresponding to the OTU. Then, the representative sequences were checked against the Maarj*AM* database (Opik et al., 2010). The unmatched representative sequences were further blasted (Altschul et al., 1990) against the National Center for Biotechnology Information (NCBI) GenBank database.^1^ Non-Glomeromycotina sequences were removed before downstream analysis. OTUs with reads fewer than 10 were also removed. All raw sequences were deposited in the Sequence Read Archive (SRA) of the NCBI database (PRJNA780785). Further, the BLAST function against the GenBank database at the NCBI was used to retrieve closely related Glomeromycotina sequences for the representative sequences of OTUs. Neighbor-joining (NJ) phylogenetic analysis was computed based on aligned sequences using the software MEGA7, with 1,000 bootstrap replicates to evaluate the support of the tree using *Henningsomyces candidus* as the outgroup to root the tree.

### Statistical Analysis

To evaluate the effect of AMF treatments on plant biomass compared with the control, the commonly used index logarithm of response ratio (lnRR) was used (Hedges et al., 1999; Oksanen et al., 2006). Specifically, the mycorrhizal growth response (MGR) of plants was calculated as ln (AM/NM), where AM is the total plant biomass of mycorrhizal plants and NM is the average of total plant biomass of non-inoculated plants. Positive MGR values indicate mutualistic relationships, and negative values indicate parasitic relationships (Ji et al., 2013; Stanescu and Maherali, 2017). Specific plant–soil feedback (PSF) was calculated as ln (Conspecific/Heterospecific), where Conspecific is the average total plant biomass of one species inoculated with Conspecific AMF and Heterospecific is the total plant biomass of the same species but inoculated with AMF from other host species. This metric could reflect whether each host species does better when inoculated with conspecific AMF (positive feedback) or with heterospecific inocula (negative feedback) (Petermann et al., 2008; Pernilla Brinkman et al., 2010; Chung et al., 2019).

All statistical analyses were conducted using R software (version 3.6.3). The model assumption of normality was tested using the Shapiro–Wilk test, and the assumption of equal variance was tested using Levene’s test. Data that did not meet the assumption were subject to log transformation. Two-way analysis of variance (ANOVA) was performed separately to test for differences in plant performance to each inoculation treatment, followed by Tukey’s HSD *post hoc* analysis when there was significance with *p* < 0.05. We used *t*-tests to test if the values of MGR and PSF significantly differed from zero.

Due to a large variation in library size, four samples with less than 5,000 reads were discarded (two samples from inoculum C and two samples from inoculated plants). To avoid bias in the remaining samples with unequal sequencing depth, we iteratively calculated the alpha diversity based on 1,000 rarefied OTU tables (rarefied to 5,283 reads) using the “estimate_richness” function in the phyloseq package (McMurdie and Holmes, 2013). One-way ANOVA was first performed to compare the diversity of AMF inocula among their natural hosts. Then, a two-way ANOVA was performed to compare the diversity of AMF-inoculated plants among host plant species and inoculation treatments followed by Tukey’s HSD *post hoc* analysis. We used PERMANOVA to test whether host plant species and inoculation treatments could explain differences in the composition of the AMF community. We further visualized these differences at a community level by non-metric multidimensional scaling (NMDS) using the vegan package (Oksanen et al., 2013). A comparison of Bray–Curtis AMF dissimilarity was also conducted to test the effect of host species and inoculum origin on AMF beta diversity. Then the linear discriminant analysis effect size (LEfSe) method was used to determine the differences in root AMF communities among inoculated plants by the microbiomeMarker R package (Segata et al., 2011; Cao, 2020).

Network analysis was used to determine the non-random interactions of the root AMF community (Barberan et al., 2012). We separated all the samples into four groups and built correlation networks for each inoculum origin separately (i.e., inoculum A, B, C, and D) to understand the co-occurrence patterns of AMF among different inoculation treatments based on the rarefied OTU table. For each network, we used the data of samples from all four plant species, while OTUs that occurred in less than half samples were removed. Briefly, the Spearman’s correlation coefficient between the pairwise OTUs was inferred using the Psych package, and we considered a relationship to be robust when *r* > |0.6| and the FDR-adjusted *p* < 0.05 (Benjamini and Hochberg, 1995). Then, a set of network and node properties were calculated with the igraph package (Csardi and Nepusz, 2005), including the indices indicating the network complexity (the number of nodes and links, links per node, average degree, average clustering coefficient, and modularity) (Banerjee et al., 2019). Subsequently, 1,000 Erdös-Rényi random networks corresponding to each of the four networks with an identical number of nodes and edges were generated to determine whether the features of observed networks were significantly different from those of random networks by one-sample *t*-test. Moreover, sub-networks for each sample were generated from networks of inoculum A, B, C, and D by preserving OTUs presented in each sample using the subgraph function following a previously described method (Ma et al., 2016). After calculating the network properties for each sub-network, linear regression was performed to explore relationships between network properties and the plant variables (MGR and PSF). Linear regression was also performed to explore relationships between the plant variables and AMF diversity, as well as abundance.

## Results

### Both Host Plant Species and Inoculum Origin Influenced Rhizosphere Arbuscular Mycorrhizal Fungi Communities

All samples yielded positive PCR products of the expected size (about 250 bp), and the rarefaction curve suggested that our sampling method captured most of the community (Supplementary Figure 1). After removing chimeras and non-AMF taxa, 165 putative AMF OTUs were affiliated to genus levels within orders of Diversisporales, Glomerales, and Paraglomerales.

**FIGURE 1:**
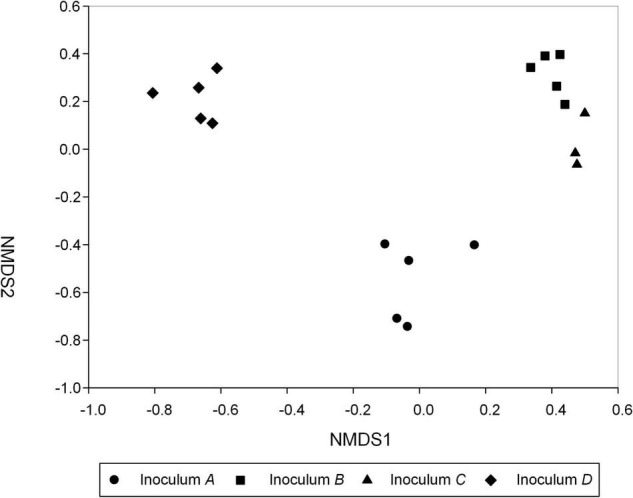
Soil AMF community structure under four inocula. Non-metric multidimensional scaling (NMDS) illustrates the differences in AMF communities for different inocula obtained from *C. korshinskii* (Inoculum A), *H. leave* (Inoculum B), *C. microphylla* (Inoculum C), and *P. annua* (Inoculum D) plants after trap culture. The value of stress was 0.038. Note that two samples of inoculum C were discarded due to low sequencing depth.

The most abundant AMF genera in the inocula A, B, C, and D were *Claroideoglomus*, *Archaeospora*, *Glomus*, and *Rhizophagus*, respectively (Supplementary Figure 2). Dramatically different species composition but similar diversity was found for the AMF communities among the four inocula (Figure 1). Specifically, PERMANOVA found a significantly different AMF species composition among the inocula of *C. korshinskii* (A), *H. leave* (B), *C. microphylla* (C), and *P. annua* (D) (*R*^2^ = 0.823, *p* < 0.001). There were an average of 60.76 ± 3.95, 65.42 ± 1.01, 61.81 ± 1.67, and 58.90 ± 1.59 AMF OTUs in inocula A, B, C, and D, respectively. No significant difference in the species richness and Shannon diversity index was detected among the four inocula (Supplementary Figure 3).

**FIGURE 2:**
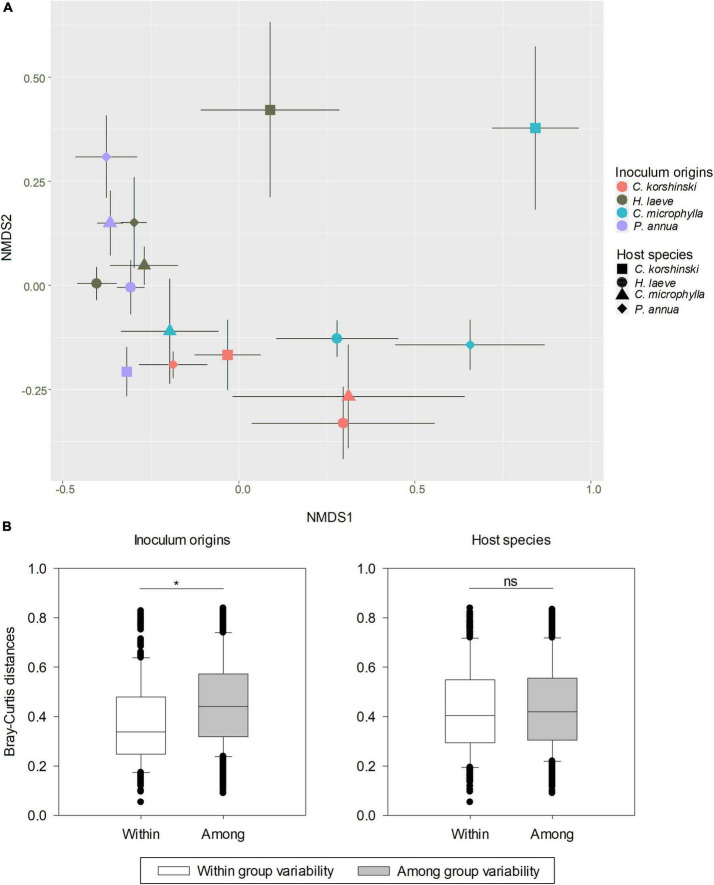
Changes in soil AMF community structure under different host species and inoculum origin. **(A)** Non-metric multidimensional scaling (NMDS) illustrates the differences in AMF communities for host plant species and inoculum origin (stress = 0.135). **(B)** Pairwise comparisons of Bray–Curtis distances among all rhizosphere AMF communities, either those associated with the same host species or the same inoculum origin (category “Within”) or those associated with two different host species or inoculum origins (category “Among”). *T*-test results showed a significant effect of inoculum origins on the dissimilarity of the AMF community.

No AMF-related structure was found in non-AMF inoculated plants. For the AMF community in the inoculated plants after harvest, 158 OTUs belonging to 10 genera were found in the roots of *C. korshinskii, H. laeve, C. microphylla*, and *P. annua* (Supplementary Figure 4). Two-way ANOVA showed that only AMF inoculation had a significant effect on the observed OTU richness (Table 1, *F* = 2.888, *p* = 0.047), while the Shannon diversity index was neither influenced by AMF inoculation nor host plant species. Nevertheless, out of six AM families, five families were significantly influenced by host species, four AM families were significantly influenced by inoculation treatments, and five families were significantly influenced by both host species and inoculation treatments (Supplementary Table 1). Besides, out of the 20 most abundant AMF OTUs, 12 OTUs were significantly influenced by host species, all 20 OTUs were significantly influenced by inoculation treatments, and 17 OTUs were significantly influenced by both host species and inoculation treatments (Supplementary Table 2). Specifically, inoculum D had the highest abundance of Diversisporaceae. Moreover, the AMF community structure of roots was significantly affected by both the host plant species (*R*^2^ = 0.091, *p* = 0.002) and their inoculum origin (*R*^2^ = 0.286, *p* < 0.001), and their interaction (*R*^2^ = 0.231, *p* < 0.001) according to PERMANOVA. NMDS analysis also confirmed that the host species and inoculum origin had strong effects on AMF community structure (Figure 2A, stress = 0.135). Even though inoculum origin, but not host species, had a more pronounced effect on AMF community dissimilarity (β diversity), the values of the Bray-Curtis distances between rhizosphere AMF communities of host plants in the same inoculum were significantly lower than those observed in different inocula (Figure 2B, *t*-test, *p* < 0.001). Likewise, LEfSe analysis found differences in AMF abundance among different inoculum origins (Supplementary Figure 5). LEfSe analysis was also performed to detect differences in AMF abundance among different host species, but no significant difference was observed among four host plant species.

**TABLE 1:** Results of two-way ANOVA and PERMANOVA showing the effects of host plant species, AMF inoculation, and their interactions on root AMF diversity, abundance (all three orders), and community structure.

	Shannon		Richness		Diversisporales		Glomerales		Paraglomerales		Community structure	
						
	F	P	F	P	F	P	F	P	F	P	R2	P
Host species	1.228	0.311	0.415	0.743	7.760	<0.001	3.851	0.016	5.347	0.003	0.091	0.002
AMF inoculation	1.717	0.178	2.888	0.047	21.705	<0.001	17.572	<0.001	0.094	0.963	0.286	<0.001
Host:AMF	0.622	0.771	1.376	0.230	4.021	0.001	4.866	<0.001	1.684	0.125	0.231	<0.001

### Significant Plant Responses to Arbuscular Mycorrhizal Fungi Inoculation

**FIGURE 3:**
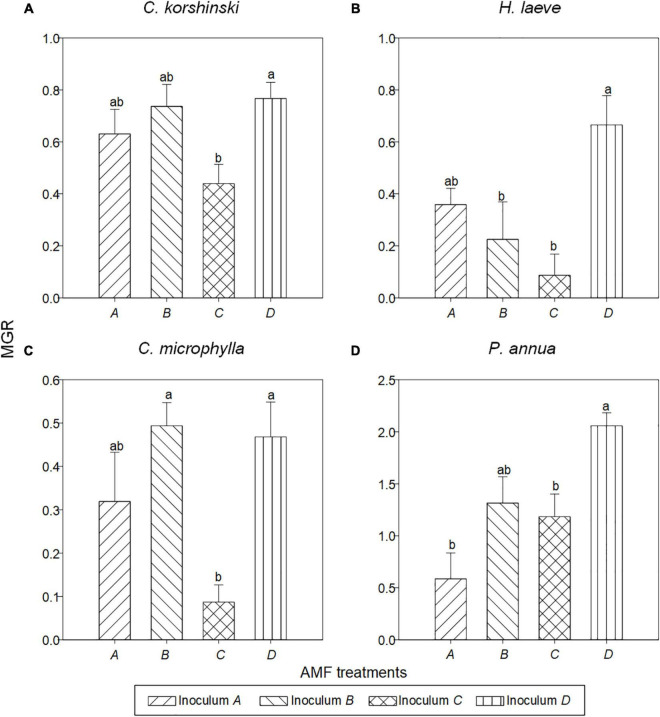
Mycorrhizal growth responses of the AMF inoculation in different plant species: **(A)** *C. korshinski*, **(B)** *H. leave*, **(C)** *C. microphylla*, and **(D)** *P. annua*. Different letters above bars indicated significant differences at *p* < 0.05 level according to Tukey’s test.

Overall, all the plant roots were well colonized by AMF (Supplementary Table 3). Host plant species (Table 2, *p* < 0.001) and AMF inoculation (*p* < 0.001) significantly affected the plant biomass and their interaction (*p* < 0.001). Generally, *C. korshinskii*, *H. laeve*, *C. microphylla*, *and P. annua* plants in the non-AMF treatment had the lowest plant biomass, and the biomass of these four species was increased by AMF by 97.6, 50.6, 46.5, and 381.1%, respectively, relative to control (Supplementary Table 1). In other words, positive MGR was found for all four species (*t*-test, *p* < 0.001) and was influenced by both the host plant (*p* < 0.001) and AMF inoculation (*p* < 0.001). Specifically, the annual grass *P. annua* had the highest MGR (mean = 1.285 ± 0.134), particularly when grown in conspecific AMF communities (mean = 2.057 ± 0.125). However, the MGR of the other three legume shrubs was lower and ranged from 0.087 to 0.767, with the highest MGR demonstrated in heterospecific AMF communities (Figure 3). Notably, plants inoculated with AMF from *P. annua* (inoculum D) had the highest MGR (mean = 0.989 ± 0.111), while inoculum C from *C. microphylla* had the lowest MGR (mean = 0.449 ± 0.093).

**TABLE 2:** Results (*p*-value) of two-way ANOVA showing the effects of host plant species, AMF inoculation, and their interactions on plant performance.

	Above-ground biomass	Below-ground biomass	Total biomass	Height	MGR	PSF	Colonization rate
Host species	<0.001	<0.001	<0.001	<0.001	<0.001	<0.001	0.054
AMF inoculation	<0.001	<0.001	<0.001	<0.001	<0.001	0.002	0.061
Host:AMF	0.027	<0.001	<0.001	<0.001	<0.001	0.037	0.063

Plant–soil feedback was also affected by both host species (*p* < 0.001) and AMF inocula (*p* = 0.002), and their interaction (*p* = 0.037) (Table 2). We detected significant feedback irrespective of AMF treatments for *C. microphylla* (*t*-test, *p* < 0.001) and *P. annua* (*t*-test, *p* < 0.001) (Supplementary Figure 6). In detail, *C. microphylla* plants grew better in heterospecific AMF communities and showed a negative PSF, while *P. annua* plants showed a positive PSF, indicating that they would grow better in conspecific AMF communities. *C. korshinskii* and *H. laeve* also showed directional feedbacks when associated with certain AMF inocula. For example, *C. korshinskii* plants grew worse when grown with AMF from *C. microphylla*, compared to conspecific AMF. However, for *H. laeve*, they would grow either similarly, better, or worse when associated with heterospecific AMF.

### Network Analysis Revealed Different Patterns of Rhizosphere Arbuscular Mycorrhizal Fungi in Different Inocula

**FIGURE 4:**
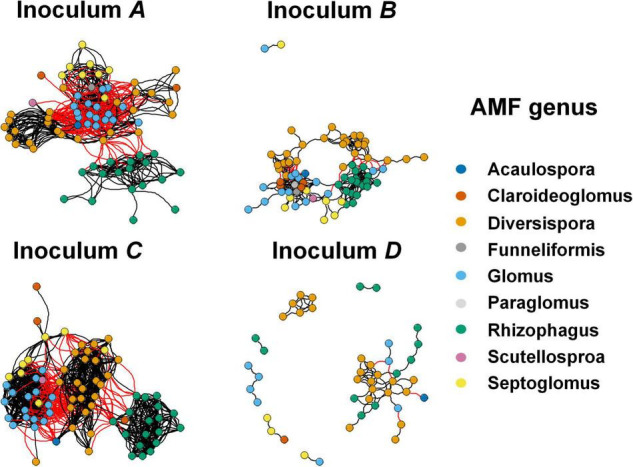
Co-occurrence network of rhizosphere AMF communities subjected to inoculum **A, B, C** and **D**. Each node represents AMF OTU, and nodes are colored based on their membership at the genus level. A black line indicates a positive interaction between two nodes, while a red line indicates a negative interaction.

**FIGURE 5:**
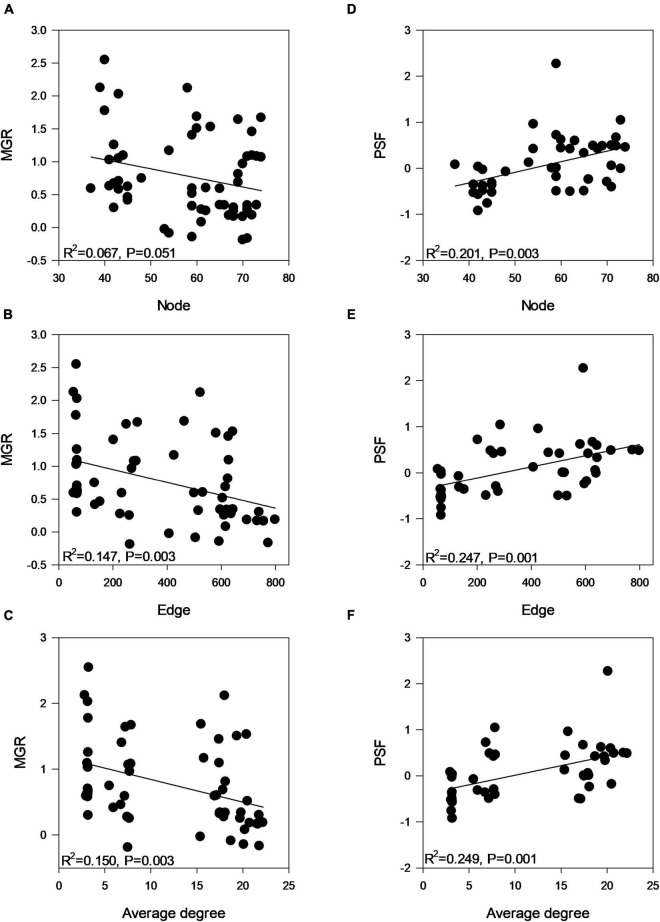
Linear regression analysis between **(A–C)** plant MGR and rhizosphere AMF network properties and **(D–F)** plant PSF and rhizosphere AMF network properties indicating the network complexity (number of nodes, edges, and average degree). Significant negative/positive relationships between network complexity and MGR/PSF were recorded.

Co-occurrence network analysis revealed different co-occurrence patterns of rhizosphere AMF communities associated with plants that were inoculated with different AMF (Figure 4). Interestingly, the network of AMF associated with inoculum D-treated plants possessed a low number of nodes, edges, average degree, corresponding to a low connectedness, while having a high level of modularity and positive correlations (Table 3). Further, we compared the network properties with the 1,000 random networks and confirmed the non-random patterns of rhizosphere AMF. Specifically, the network of roots inhabiting AMF subjected to inoculum D had a lower network diameter and average path distance than the random works. The other three networks (inoculum A, B, C, and D) had the opposite patterns. All four networks had higher modularity and module number compared to that of random networks. After generating sub-networks for each sample corresponding to their affiliated inocula, the average degree of a network which indicated the network complexity was calculated. Linear regression showed significant negative relationships between the number of nodes, edges, average degree, and MGR (Figures 5A–C, *p* = 0.051, 0.003, and 0.003), while positive relationships were revealed between the network properties and PSF (Figures 5D–F, *p* = 0.003, 0.0011, and 0.001). In addition, neither AMF alpha diversity nor beta diversity was found to be related to plant performance. Family-level AMF abundance was not related to plant properties either. However, among the 20 most abundant AMF OTUs, the abundance of two *Diversispora* OTUs that were identified as indicators of inoculum D by LEfSe was found to be significantly and positively related to plant MGR (Supplementary Figure 7).

**TABLE 3:** AMF network properties of original and random networks.

		Node	Edge	Edges per node	Average degree	Average clustering coefficient	Average path distance	Density	Diameter	Connectedness	Modularity	Module number	Proportion of positive correlations
Original network	*A*	74	645	8.716	17.432	0.765	2.317	0.239	6	1	0.369	4	77.92%
	*B*	75	294	3.920	7.840	0.605	3.532	0.106	10	0.947	0.601	6	88.95%
	*C*	73	801	10.973	21.945	0.790	2.113	0.305	6	1	0.459	4	87.38%
	*D*	45	69	1.533	3.067	0.482	2.878	0.070	7	0.356	0.616	10	93.7%
Random network	*A*	74	645	8.716	17.432	0.239	1.772	0.239	3.000	1	0.157	3.928	
	*B*	75	294	3.920	7.840	0.105	2.297	0.106	4.070	0.1	0.282	5.626	
	*C*	73	801	10.973	21.945	0.305	1.696	0.305	2.784	1	0.128	3.584	
	*D*	45	69	1.533	3.067	0.069	3.295	0.070	7.231	0.916	0.473	7.792	

*Random networks with the same numbers of nodes and edges for each of the four networks were generated, whose network properties were calculated as the average value of the 1,000 networks.*

## Discussion

Our primary hypothesis was to assess whether AMF inoculation could promote plant growth and whether AMF conditioned by conspecific or heterospecific host species would differ in the promotion. We also wanted to link plant response (MGR and PSF) with the root AMF community to identify whether this response was driven by AMF or not. Overall, our data showed that all four studied species had a positive MGR, indicating the promotion of plant biomass by AMF, while the specific PSF was more complicated and depended on the combination of host species and fungal inoculum. Linear regression showed a significant negative correlation between the rhizosphere AMF network complexity and the MGR.

### Plant Mycorrhizal Responses Depend on the Combination of Host Plant Species and Arbuscular Mycorrhizal Fungi

In this study, all four species showed a positive MGR, which means the application of AMF inoculum improved the plant biomass. It has been well-established that plants modify their associated soil microbiota and result in the accumulation of species-specific pathogens, mutualisms, and other microbes (Callaway et al., 2004; Berendsen et al., 2012; Jain et al., 2020). Living an obligate lifestyle, AMF only use the carbon provided by the plant directly (Jiang et al., 2017), thus their relationship was more intimate. In the desert system, where the original inoculum was collected, the symbiosis between plant and AMF should be highly efficient to survive, thus the positive effects induced by AMF inoculation were not surprising, as reported in other studies using indigenous AMF and grown in the desert soil or the desert environment (Requena et al., 2001; Bashan et al., 2009; Zhang et al., 2012, 2019; Harris-Valle et al., 2018).

Our second hypothesis was that plants would grow better under conspecific inocula. This was true only for the annual grass *P. annua*, but not for the other three legume shrubs. The reason may be because inoculum D, which was conditioned by the annual grass *P. annua*, was the most beneficial, while the other three inocula were inferior in promoting plant growth. Such variation in plant responses to AMF was not surprising considering the species-specific effect between plants and AMF. However, the great benefits of inoculum D were of great interest. We know that host plant identity and the functional group were the major determinants that shape the AMF community (Martinez-Garcia et al., 2015; Davison et al., 2020). A previous study found that grass allocated more carbon to its AMF partners than the legume using the C^13^ labeling technique (Ladygina and Hedlund, 2010). Weber et al. (2019) also found that grasses were associated with a high abundance of rhizophilic AMF that primarily colonized roots with a high allocation to intraradical hyphae. Other studies reported that the fast-growing annual grass favored fine AMF to complete their life cycle in a relatively short period of time (Thippayarugs et al., 1999; Hilbig and Allen, 2015; Phillips et al., 2019). While perennial shrubs could form the island of fertility (Bonanomi et al., 2007; Blaser et al., 2014; Lan et al., 2021) and may favor the indirect help provided by AMF, such as stress tolerance. Taken together, under the selectivity of *P. annua*, the inoculum D may contain specific AMF communities which could facilitate AMF colonization and promote plant growth at an early stage.

To detangle the effects of different AMF guilds, we need to fully explore the AMF community composition. In the past, researchers relayed on spore morphology to characterize AMF communities, but this method required skilled experts and was subjective (Bai et al., 2013). Later, there were other ways to characterize AMF communities either quantitatively or qualitatively, such as the fatty acid (FA) signature method, the restriction fragment length polymorphism (RFLP), and the denaturing gradient gel electrophoresis (DGGE) method (Ma et al., 2005; Dickie and FitzJohn, 2007; Olsson and Lekberg, 2022). However, only the development of sequencing methods in recent decades provided a perfect tool to characterize AMF communities. Using the high-throughput sequencing, Varela-Cervero et al. (2015) found that *Glomus* species were mainly detected in roots, while *Diversispora* were detected more in the extraradical hyphae. Babalola et al. (2022) found that AMF of different guilds responded differently to nitrogen fertilization. To elucidate certain AMF taxa or group effects on plant performance and to explain the great beneficial effects of inoculum D, Illumina sequencing was performed to explore the AMF community within the inoculated plant roots.

The PERMANOVA results confirmed the effects of inoculation treatments on root-associated AMF communities within the inoculated plants. LEfSe analysis further revealed that Glomeraceae was more abundant within inoculum C, while Diversisporaceae was more abundant within inoculum D. Three indicator OTUs of *Diversispora* were also revealed for inoculum D, and two of them were positively correlated to plant MGR. As described by Weber et al. (2019), the Diversisporaceae belonging to the edaphophilic guild could improve plant nutrient uptake via extensive extraradical hyphae, while the Glomeraceae AMF belonging to the rhizophilic AMF would preferentially allocate more carbon to colonized roots. Recently, a previous study reported a positive correlation between plant performance and the abundance of *Diversispora* AMF (Guo et al., 2021). Moreover, a recent meta-analysis reported that the AMF benefits on plants varied among species and taxonomic group, with Diversisporales being the most beneficial to plants (Marro et al., 2022). Based on these findings, we could primarily attribute the great benefits of inoculum D to the high abundance of Diversisporaceae.

### Plant Responses Are Linked to the Rhizosphere Arbuscular Mycorrhizal Fungi Network Complexity

We assumed that the magnitude of plant responses was the net consequence of AMF community interactions. Unexpectedly, neither the alpha diversity nor the beta diversity of the rhizosphere AMF community was related to the plant’s MGR and PSF. Since the plant–AMF interaction was influenced by the development stage of plants (Lü et al., 2020; Gutiérrez-Núñez et al., 2022), we assumed it may take time to establish a strong relationship between them. In addition, sometimes AMF identity but not the AMF diversity may be more important (Vogelsang et al., 2006; Liu et al., 2020). In this study, AMF involved in the network and their interactions were important. Specifically, the network complexity of the rhizosphere AMF community was represented by the number of nodes, edges, and average degree of the network, as revealed in the previous studies (Bennett et al., 2018; Banerjee et al., 2019; Li et al., 2020), and was found to be negatively related to the strength of plant MGR and positively related to the plant PSF.

Following developments in sequencing technology, we know that microorganisms do not exist in isolation, but form complex ecological associations amongst each other (Strogatz, 2001; Barberan et al., 2012). Take AMF for example, previous studies have documented the complementary, competition, facilitation, and suppression interactions among the rhizosphere AMF communities (Koide, 2008; Koch et al., 2012; Symanczik et al., 2015; Janouskova et al., 2017; Martignoni et al., 2020). In our study, network analysis revealed different co-occurrence patterns of AMF among inoculation treatments. Compared with the other three networks, the network of AMF in inoculum D contained less nodes and edges, and had a lower average degree but high modularity. It has been reported that the complexity of microbial networks was related to environmental changes (Fernandez-Gonzalez et al., 2020; Lin et al., 2021; Ma et al., 2021; Qiu et al., 2021; Liu et al., 2022). Specifically, a recent study reported that complex microbial networks were conducive for microbes to adapt to adverse environments, such as drought and invasion (Fu et al., 2022; Gao et al., 2022; Wang et al., 2022). And in our study, the network properties of AMF were also linked with plant responses to inoculation. Specifically, the fewer AMF OTUs involved in the network with low complexity, the more beneficial they were to plant growth, such as the inoculum D. This contradicted a previous study that found positive relationships between bacterial network complexity and maize yield (Tao et al., 2018). The more beneficial effects of inoculum D on plant growth might be due to the dramatically higher proportion of positive correlations among AMF species in inoculum D relative to other inocula (Table 3). This higher proportion of positive correlations in inoculum D indicated more complementary metabolisms, which enhanced their beneficial effects. In addition, higher network modularity was detected in inoculum D relative to other inocula, which may also contribute to their beneficial effects on plant growth (Delmas et al., 2018; Banerjee et al., 2019; Lasa et al., 2022).

## Conclusion

Overall, our study used native AMF in the desert to set conspecific or heterospecific combinations of the plant and fungi and inoculated plants in the greenhouse. In this study, we confirmed the positive effects of AMF inoculation on plant biomass and revealed context-dependent plant–soil feedback effects. Though the effect of plant–soil feedback may not essentially predict the real consequences in the field (Yelenik and Levine, 2011), particularly considering the extreme drought in the desert, our results emphasized the importance of plant–AMF interactions in the restoration and further addressed the necessity to select proper afforestation species and AMF sources, especially when multiple plant species were involved.

## Data Availability Statement

The datasets presented in this study can be found in online repositories. The names of the repository/repositories and accession number(s) can be found in the article/Supplementary Material.

## Author Contributions

BJ and XW conceived and designed the study. XG and PW carried out the experiment. XG analyzed the data and wrote the first manuscript. BJ and YL reviewed and edited the manuscript. All authors contributed to the article and approved the submitted version.

## Conflict of Interest

The authors declare that the research was conducted in the absence of any commercial or financial relationships that could be construed as a potential conflict of interest.

## Publisher’s Note

All claims expressed in this article are solely those of the authors and do not necessarily represent those of their affiliated organizations, or those of the publisher, the editors and the reviewers. Any product that may be evaluated in this article, or claim that may be made by its manufacturer, is not guaranteed or endorsed by the publisher.

## Footnotes

1
https://www.ncbi.nlm.nih.gov/gene
