# Supplementary material for: Specific Plant Mycorrhizal Responses Are Linked to Mycorrhizal Fungal Species Interactions
Source: Front Plant Sci. 2022 Jun 10;13:930069. doi: 10.3389/fpls.2022.930069 (PMC9226604; doi:10.3389/fpls.2022.930069)
Supplement: Supplementary file 1 [file Data_Sheet_1.docx]

Supplementary Material

**Supplementary Figure S1.** Rarefaction curves of AMF species richness along the number of sequences obtained from the 4 inocula (A) and rhizospheres of 4 inoculated host plant species (B).


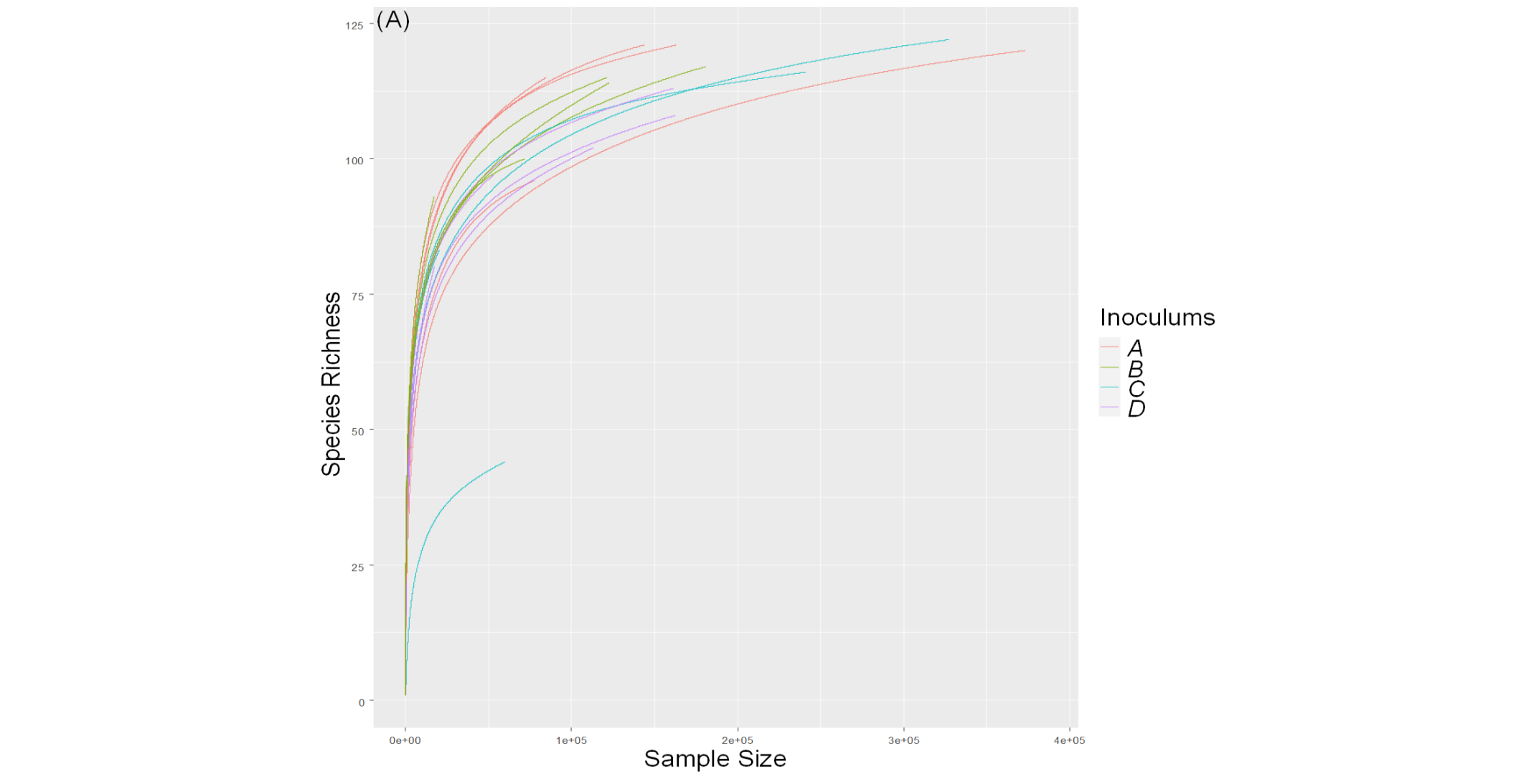

**Supplementary Figure S2.** Relative abundance of AMF found in the four inocula at family and genus level.







**Supplementary Figure S3.** Species richness (left) and Shannon diversity (right) of AMF associated with 4 inocula.


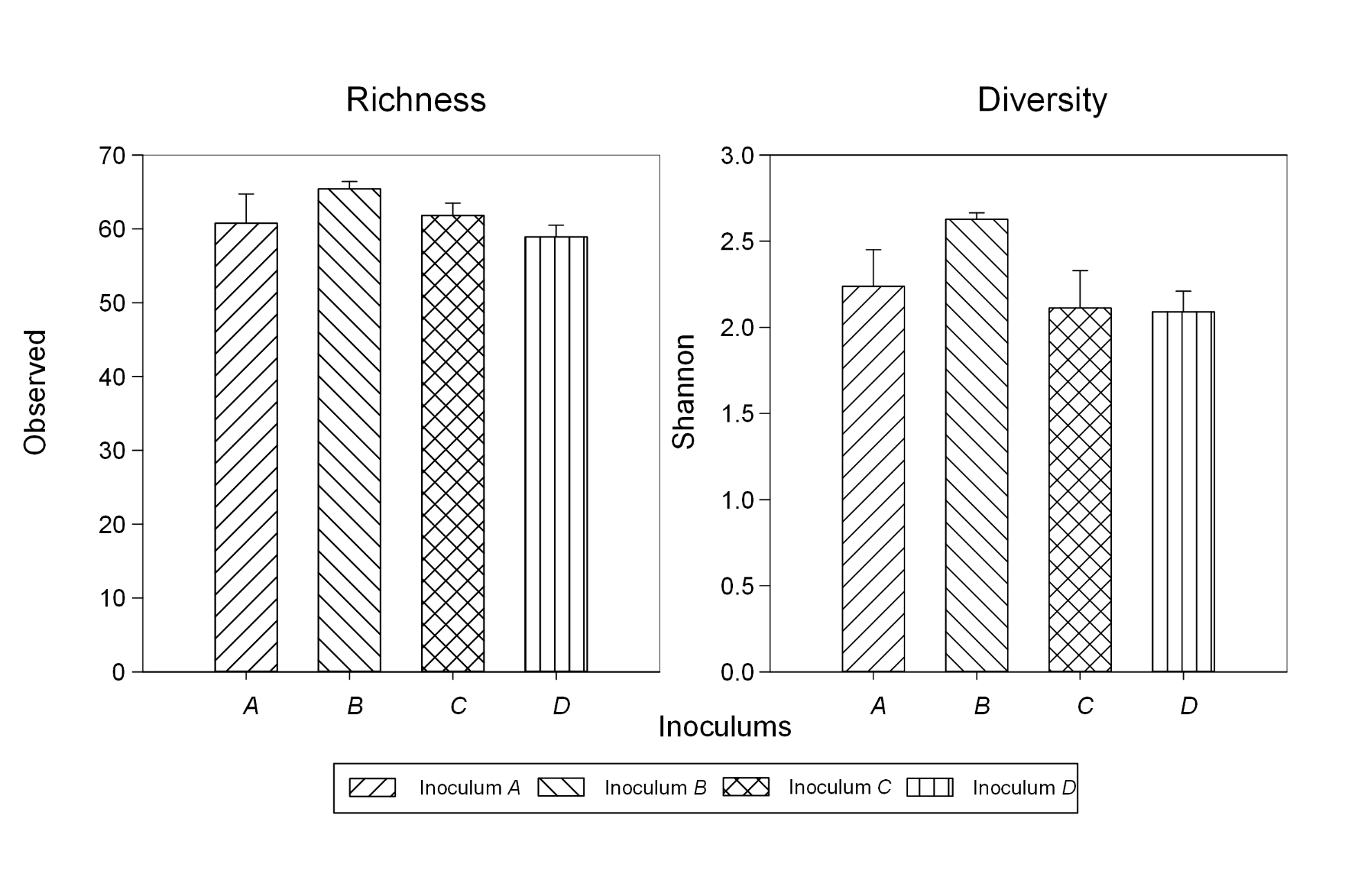


**Supplementary Figure S4.** Relative abundance of AMF genus associated with four inoculated species.


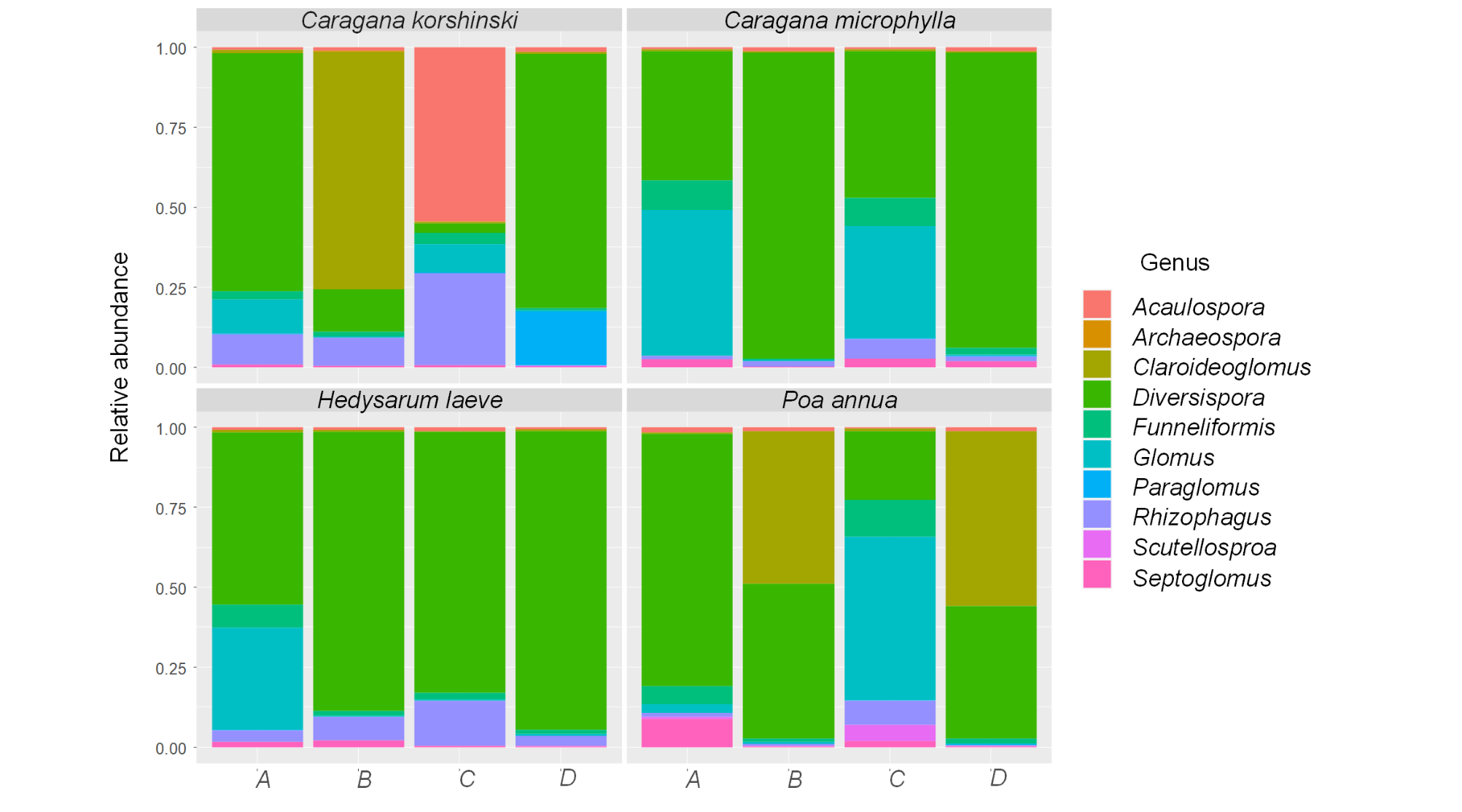


**Supplementary Figure S5.** Linear discriminant analysis effect size (LEfSe) showing the differences in abundances of rhizosphere AMF among inoculated plants at multiple taxonomical levels when harvested. Significant differences in AMF abundances were found only among the AMF inoculation treatments but not for host plant species. Inoculum *C*-treated plants had a high abundance of Glomerales, while plants treated with inoculum *D* had a high abundance of Diversisporales.


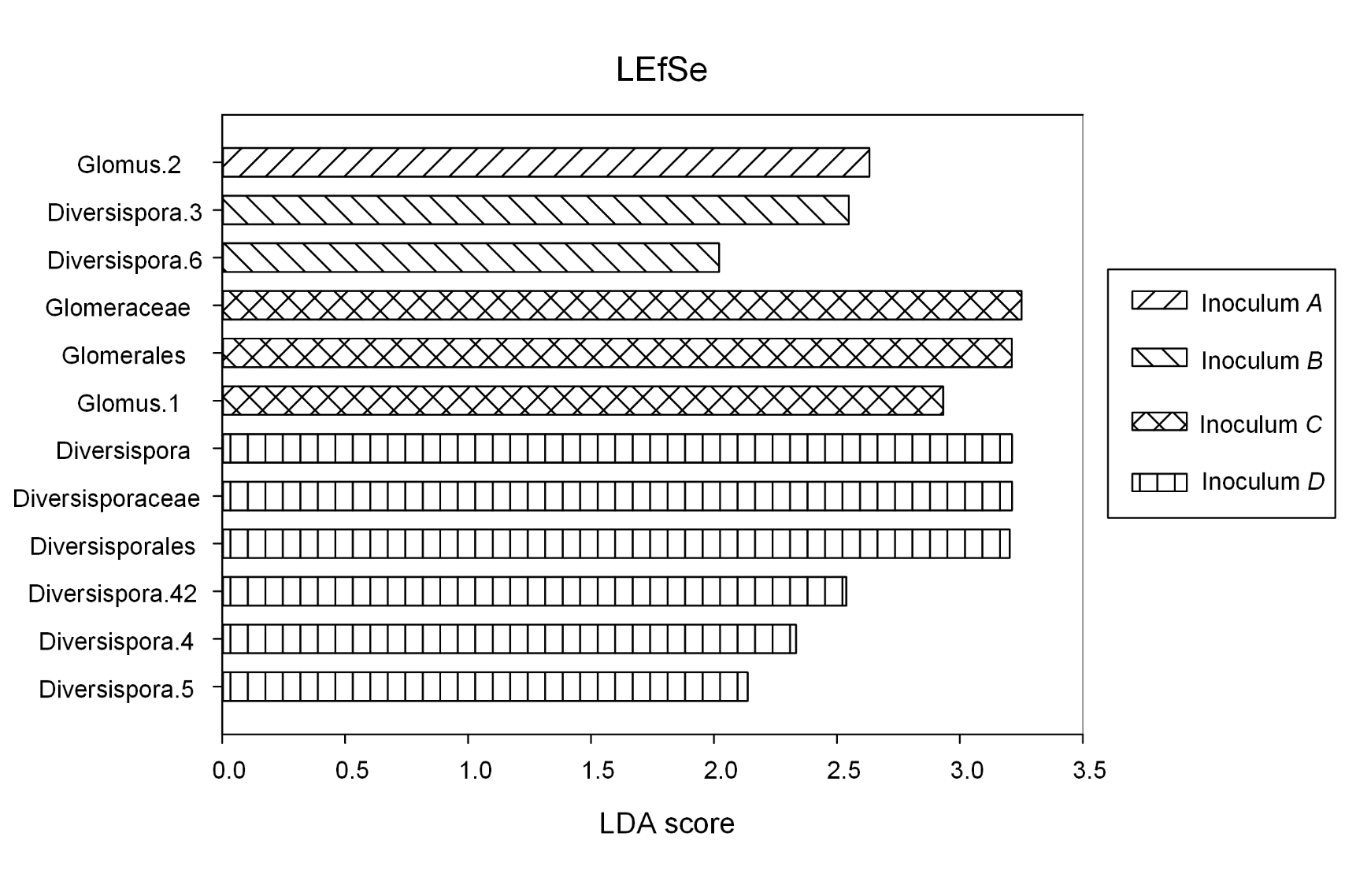


**Supplementary Figure S6** Specific plant-soil feedbacks of the AMF inoculation under *C. korshinsk, H. leave*, *C. microphylla* and *P. annua* plants. Significance was determined by t-tests to test if the values of PSF were significantly differed from zero ("**" P ≤ .01, "*" P ≤ .05, "ns" P > .05).


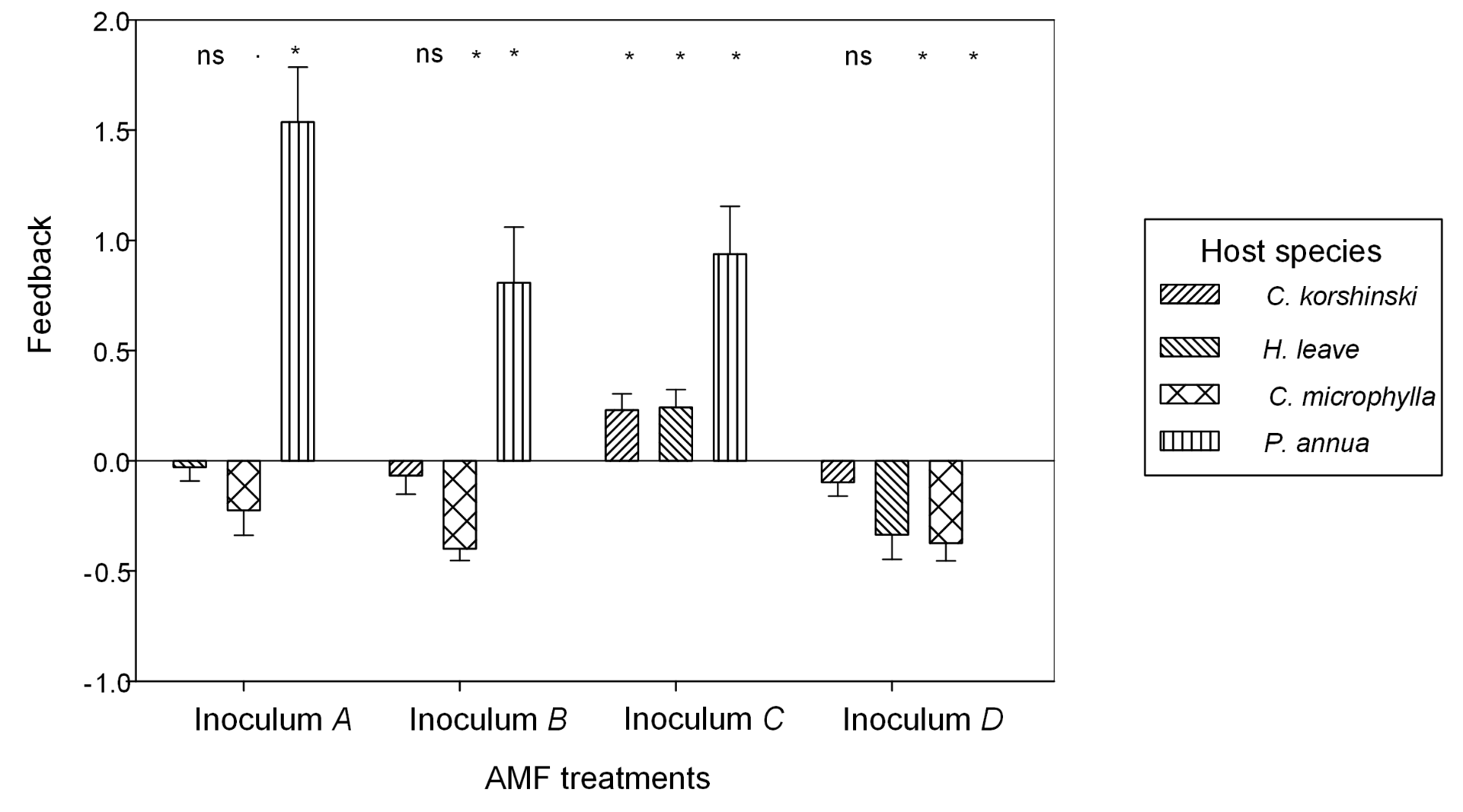


**Supplementary Figure S7** Significant and positive relationships between plant MGR and the abundance of two *Diversispora* OTUs that identified as indicators of inoculum *D*.





**Supplementary Table S1** Results of two-way ANOVA showing the effects of host plant species, AMF inoculation and their interactions on root AMF abundance at the family level.

|  | Host | AMF | Host:AMF |
| --- | --- | --- | --- |
| Acaulosporaceae | 0.491 | 0.001 | 0.006 |
| Diversisporaceae | 0.000 | 0.000 | 0.001 |
| Gigasporaceae | 0.011 | 0.089 | 0.035 |
| Claroideoglomeraceae | 0.000 | 0.000 | 0.000 |
| Glomeraceae | 0.027 | 0.000 | 0.000 |
| Paraglomeraceae | 0.003 | 0.963 | 0.125 |

**Supplementary Table S2** Results of two-way ANOVA showing the effects of host plant species, AMF inoculation and their interactions on root AMF abundance of the most abundant 20 OTUs.

|  | Host | AMF | Host:AMF |
| --- | --- | --- | --- |
| OTU_1 | 0.002 | 0.000 | 0.000 |
| OTU_2 | 0.010 | 0.000 | 0.000 |
| OTU_3 | 0.002 | 0.000 | 0.000 |
| OTU_4 | 0.134 | 0.000 | 0.001 |
| OTU_5 | 0.164 | 0.000 | 0.000 |
| OTU_6 | 0.000 | 0.000 | 0.000 |
| OTU_7 | 0.000 | 0.000 | 0.021 |
| OTU_9 | 0.530 | 0.006 | 0.003 |
| OTU_10 | 0.715 | 0.000 | 0.000 |
| OTU_11 | 0.542 | 0.049 | 0.062 |
| OTU_12 | 0.003 | 0.000 | 0.144 |
| OTU_18 | 0.003 | 0.000 | 0.001 |
| OTU_19 | 0.320 | 0.000 | 0.001 |
| OTU_21 | 0.095 | 0.013 | 0.017 |
| OTU_23 | 0.000 | 0.000 | 0.010 |
| OTU_69 | 0.001 | 0.000 | 0.015 |
| OTU_86 | 0.213 | 0.004 | 0.242 |
| OTU_150 | 0.003 | 0.000 | 0.000 |
| OTU_227 | 0.001 | 0.000 | 0.000 |
| OTU_256 | 0.000 | 0.000 | 0.007 |

**Supplementary Table S3** Plant variables under different AMF inoculation treatments.

| Hosts | Inoculation treatments | Above-ground biomass(g) | se | Below-ground biomass(g) | se | Total biomass(g) | se | Height(cm) | se | MGR | se | PSF | se | Colonization rate(%) | se |
| --- | --- | --- | --- | --- | --- | --- | --- | --- | --- | --- | --- | --- | --- | --- | --- |
| *Caragana korshinski* | *A* | 0.173 | 0.018 | 0.088 | 0.008 | 0.260 | 0.023 | 15.117 | 1.558 | 0.631 | 0.094 |  |  | 78.516 | 8.107 |
|  | *B* | 0.196 | 0.016 | 0.091 | 0.008 | 0.287 | 0.023 | 20.443 | 1.335 | 0.736 | 0.085 | -0.067 | 0.085 | 82.672 | 5.511 |
|  | *C* | 0.123 | 0.014 | 0.089 | 0.008 | 0.212 | 0.016 | 12.533 | 1.451 | 0.439 | 0.074 | 0.230 | 0.074 | 93.394 | 3.087 |
|  | *D* | 0.198 | 0.015 | 0.094 | 0.007 | 0.292 | 0.018 | 21.950 | 0.877 | 0.767 | 0.062 | -0.098 | 0.062 | 87.687 | 3.135 |
|  | *CK* | 0.054 | 0.006 | 0.080 | 0.006 | 0.133 | 0.011 | 4.947 | 0.354 |  |  |  |  |  |  |
| *Hedysarum laeve* | *A* | 0.107 | 0.009 | 0.082 | 0.006 | 0.189 | 0.012 | 16.297 | 0.936 | 0.359 | 0.062 | -0.030 | 0.062 | 85.762 | 3.247 |
|  | *B* | 0.105 | 0.018 | 0.076 | 0.013 | 0.180 | 0.031 | 15.100 | 0.783 | 0.225 | 0.145 |  |  | 81.900 | 2.799 |
|  | *C* | 0.073 | 0.008 | 0.073 | 0.007 | 0.146 | 0.012 | 14.907 | 0.861 | 0.087 | 0.081 | 0.242 | 0.081 | 83.093 | 0.483 |
|  | *D* | 0.151 | 0.018 | 0.117 | 0.017 | 0.268 | 0.032 | 18.817 | 0.764 | 0.665 | 0.112 | -0.336 | 0.112 | 78.646 | 3.188 |
|  | *CK* | 0.042 | 0.003 | 0.087 | 0.005 | 0.130 | 0.007 | 12.277 | 0.405 |  |  |  |  |  |  |
| *Caragana microphylla* | *A* | 0.181 | 0.017 | 0.090 | 0.009 | 0.270 | 0.026 | 18.633 | 1.914 | 0.319 | 0.113 | -0.225 | 0.113 | 87.223 | 4.072 |
|  | *B* | 0.188 | 0.008 | 0.122 | 0.011 | 0.309 | 0.016 | 19.260 | 0.813 | 0.494 | 0.053 | -0.400 | 0.053 | 88.099 | 0.738 |
|  | *C* | 0.132 | 0.006 | 0.073 | 0.004 | 0.205 | 0.008 | 15.257 | 1.006 | 0.087 | 0.039 |  |  | 91.181 | 0.693 |
|  | *D* | 0.200 | 0.015 | 0.106 | 0.008 | 0.306 | 0.021 | 22.163 | 0.516 | 0.468 | 0.080 | -0.374 | 0.080 | 82.984 | 2.785 |
|  | *CK* | 0.082 | 0.010 | 0.104 | 0.008 | 0.186 | 0.018 | 7.943 | 0.670 |  |  |  |  |  |  |
| *Poa annua* | *A* | 0.091 | 0.021 | 0.052 | 0.014 | 0.144 | 0.033 | 18.790 | 2.041 | 0.585 | 0.249 | 1.536 | 0.249 | 81.591 | 7.181 |
|  | *B* | 0.179 | 0.030 | 0.099 | 0.018 | 0.279 | 0.048 | 26.490 | 2.253 | 1.314 | 0.253 | 0.807 | 0.253 | 63.182 | 1.248 |
|  | *C* | 0.161 | 0.037 | 0.079 | 0.014 | 0.240 | 0.044 | 22.390 | 2.869 | 1.183 | 0.217 | 0.938 | 0.217 | 86.594 | 5.121 |
|  | *D* | 0.338 | 0.033 | 0.173 | 0.032 | 0.511 | 0.057 | 32.665 | 1.247 | 2.057 | 0.125 |  |  | 83.250 | 6.241 |
|  | *CK* | 0.048 | 0.013 | 0.014 | 0.004 | 0.061 | 0.016 | 6.569 | 1.867 |  |  |  |  |  |  |
